# Hypothermic Circulatory Arrest in Median Sternotomy Hemorrhage During Redo Aortic Surgery

**DOI:** 10.21470/1678-9741-2022-0164

**Published:** 2023-09-11

**Authors:** Tiansheng Tang, Changjuan Wu, Jianshi Liu, Kaitao Jian, Wei Liu, Weiyong Sheng

**Affiliations:** 1 Department of Cardiovascular Surgery, The First Affiliated Hospital of Wannan Medical College (Yijishan Hospital of Wannan Medical College), Wuhu, Anhui, People’s Republic of China; 2 Department of Pharmacy, Wannan Medical College, Wuhu, Anhui, People’s Republic of China; 3 Department of Cardiovascular Surgery, DeltaHealth Hospital, Shanghai, People’s Republic of China

**Keywords:** Sternotomy, Superior Vena Cava, Catheterization, Intratracheal Intubation, Drainage, Patient Discharge

## Abstract

**Introduction:**

This study summarizes the clinical data of patients who developed sternotomy hemorrhage during redo aortic surgery and analyzes the clinical experience of using hypothermic circulatory arrest.

**Methods:**

We retrospectively analyzed the medical records of patients who developed sternotomy hemorrhage during redo aortic surgery from May 2018 to August 2021. General anesthesia with single-lumen tracheal intubation was used. Femoral artery, vein, and superior vena cava cannulation were used if cardiopulmonary bypass was required according to the situation, and right superior vein or apical cannulation was selected for left heart drainage.

**Results:**

A total of 11 patients were enrolled in this study, comprising nine males and two females, with an average age of 44.3±16.7 years. All cases were successfully completed without cerebrovascular complications or paraplegia. Two patients died during hospitalization, two patients died during the follow-up after discharge, and the remaining patients are recovering well.

**Conclusion:**

The femoral-femoral bypass with hypothermic circulatory arrest technique is a safe and reliable method to use in cases of sternotomy hemorrhage during redo aortic surgery.

## INTRODUCTION

**Table:** 

Abbreviations, Acronyms & Symbols				
AAA	= Ascending aortic aneurysms		DM	= Diabetes mellitus
AAR	= Ascending aortic repair		F	= Female
AD	= Aortic dissection		HCA	= Hypothermic circulatory arrest
AI	= Aortic insufficiency		HF	= Heart failure
AV	= Aortic valve		M	= Male
AVR	= Aortic valve replacement		MHCA	= Moderate hypothermic circulatory arrest
BMI	= Body mass index		MI	= Mitral insufficiency
CPB	= Cardiopulmonary bypass		MVR	= Mitral valve replacement
CTA	= Computed tomography angiography		TEVAR	= Thoracic endovascular aortic repair
DHCA	= Deep hypothermic circulatory arrest			

With advancement of cardiac surgical techniques and increased life expectancy, the number of patients who may require redo aortic surgery is increasing. Due to postoperative changes in tissue adhesions, anatomical structures, and cardiac physiologic functional status, redo aortic surgery remains a great challenge for cardiovascular surgeons^[1,2]^. There are few specific reports about how to deal with aortic hemorrhage quickly and effectively during sternotomy in redo aortic surgery. This study summarizes clinical data of patients with sternotomy hemorrhage in redo aortic surgery, as well as the relevant clinical experience with femoral-femoral bypass and hypothermic circulatory arrest.

## METHODS

**Fig. 1:**
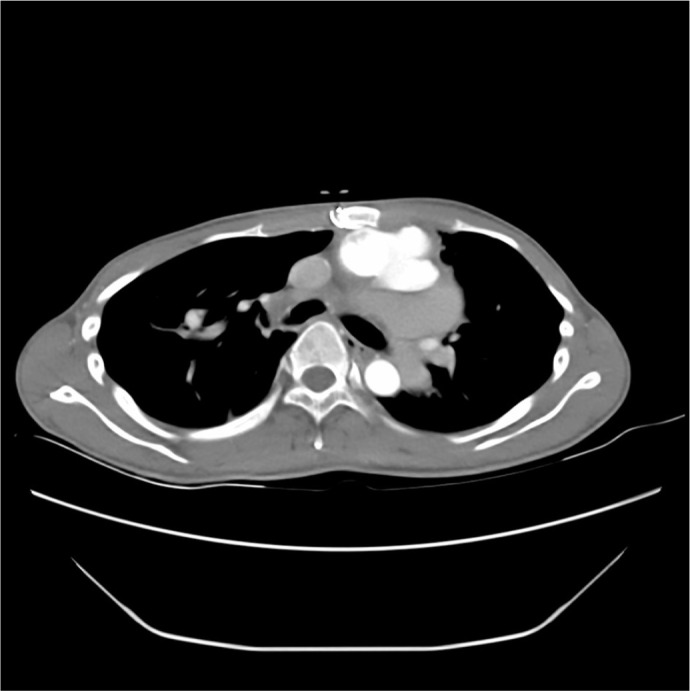
Preoperative computed tomography angiography tridimensional image depicting aortic pseudoaneurysm adherent to the sternum.

We selected a total of 11 patients treated at the Department of Cardiovascular Surgery of Shanghai DeltaHealth Hospital who underwent redo aortic surgery and developed sternotomy hemorrhage from May 2018 to August 2021 (Table 1). All cases were diagnosed based on clinical manifestations, electrocardiogram, echocardiography, and preoperative computed tomography angiography (CTA) (Figure 1), and femoral arteriovenous ultrasonography ensured the safety of femoral cannulation. The indications for redo aortic surgery are given in Table 1. This study was conducted in compliance with the tenets of the Declaration of Helsinki and was approved by the Ethics Committee of Shanghai DeltaHealth Hospital (SDH (2018) KYLWPJ 001). All patients provided written informed consent for using their clinical data for scientific presentations or publications.

**Table 1:** Patients’ characteristics.

No.	Sex	Age (years)	BMI	Comorbidities	Previous surgery	Postoperative year	Reason for redo surgery
1	F	64	20.6	None	AVR	20	AV dysfunction
2	M	25	15.1	Cerebral infarction	Bentall procedure	7	Perivalvular leak, aortic pseudoaneurysm, HF
3	M	31	24.4	Hypertension	Bentall procedure	7	Aortic pseudoaneurysm
4	M	61	22.7	Cerebral infarction	AAR	7	Aortic pseudoaneurysm, MI, AI
5	F	46	26.1	Hypertension, DM	AAR	0.7	Chronic AD
6	M	55	21.6	Hypertension	AAR + semiarch replacement	12	Aortic pseudoaneurysm
7	M	66	24.3	Hypertension	AAR + semiarch replacement	9	AAA, AI, HF
8	M	27	25.7	Hypertension	Bentall procedure	4	AD + aortic arch aneurysm
9	M	26	17.3	None	Bentall procedure	0.2	Aortic pseudoaneurysm
10	M	30	18.1	None	AAR + arch replacement	0.5	Aortic pseudoaneurysm repair
11	M	56	24.9	Hypertension	AAR+TEVAR	14	Chronic AD

AAA=ascending aortic aneurysms; AAR=ascending aortic repair; AD=aortic dissection; AI=aortic insufficiency; AV=aortic valve; AVR=aortic valve replacement; BMI=body mass index; DM=diabetes mellitus; F=female; HF=heart failure; M=male; MI=mitral insufficiency; TEVAR=thoracic endovascular aortic repair

### Surgical Technique

**Fig. 2:**
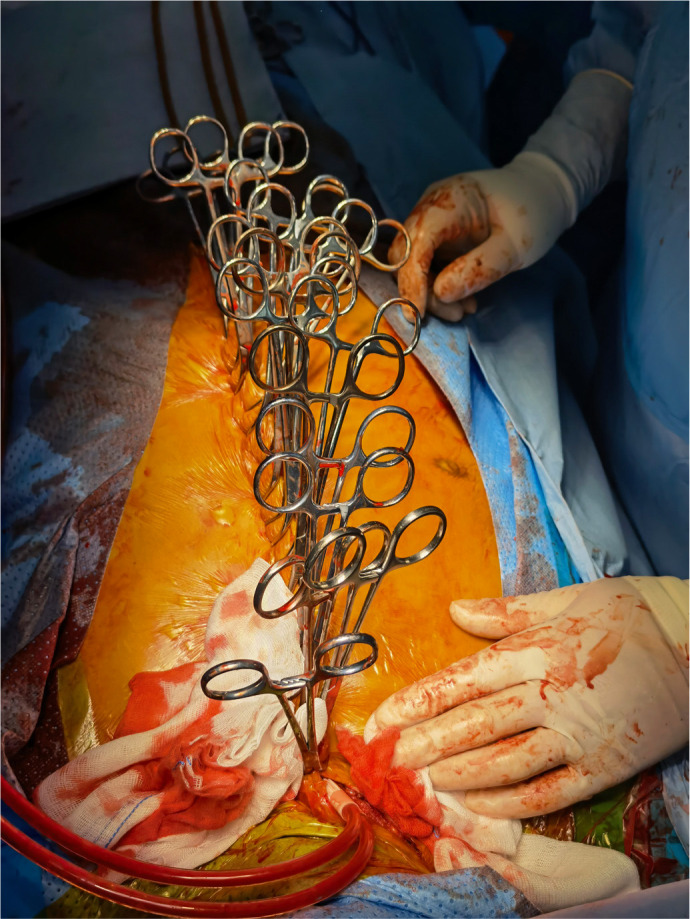
Sternum clamped with towel forceps on both sides to control hemorrhage.

Right femoral vessels were routinely prepared and exposed with a groin incision for percutaneous access, and heparin sodium (3 mg/kg) was injected into the central vein for systemic anticoagulation. An arterial cannula was inserted through the right femoral artery, and a venous drainage tube was inserted through the right femoral vein to establish cardiopulmonary bypass (CPB). Preoperative CTA demonstrated that aortic rupture during sternotomy could not be avoided in some patients (Figure 1); in these cases, the external transfer machine was directly cooled to 25ºC, and a median sternotomy was performed. For patients with aortic valve dysfunction prior to surgery, the apex of the heart was exposed through a left intercostal incision and a 3-0 PROLENE™ line with a felt suture purse for transapical left ventricular drainage. In other patients, aortic bleeding during sternotomy was possible but not inevitable. These patients underwent free femoral arteriovenous cannulation prior to sternotomy in preparation for possible sternotomy hemorrhage. In case of arterial hemorrhage during sternotomy in both patient types, the sternum was clamped with towel forceps on both sides of the sternum to control the hemorrhage (Figure 2), and CPB was started quickly. Rapid infusion and cooling were performed, with an ice cap on the head, and the head in the down position. Based on the size of the aortic crevasses and the degree of difficulty of the surgical repair, either moderate hypothermic circulatory arrest (MHCA) or deep hypothermic circulatory arrest (DHCA) was performed. The aortic rupture was sutured, or the aortic crevasse was blocked with a balloon catheter. If the hemorrhage could not be controlled by the abovementioned methods, the aortic root was freed and blocked; if freeing the aortic root was difficult, distal aortic anastomosis was performed by DHCA, then the artificial vessel was blocked after anastomosis. Once hemorrhage was controlled, circulation was restored, and a drainage tube was placed in the right upper pulmonary vein or at the apex of the heart. For mitral valve surgery, a superior vena cava cannula was routinely placed. For patients who required aortic arch replacement during surgery, routine right innominate artery cannulation was selected for cerebral perfusion. Intraoperative left common cervical cerebral perfusion was prepared depending on cerebral oxygenation. The specific surgical procedures performed on the 11 study patients are described in Table 2.

**Table 2:** Patients’ surgical methods, blood component transfusions, and outcomes.

No.	Surgical options	HCA time (min)	Rectal temperature (ºC)	Red blood cells (U)	Blood plasma (ml)	Blood platelet (U)	Complication	Follow-up
1	AVR	4	21.1	10	300	0	None	Alive
2	Cabrol procedure	2	24.2	2	0	0	Malignant arrhythmia	Dead
3	Bentall procedure	16	18.6	2	600	0	None	Alive
4	MVR + Wheat procedure	25	17.8	2	0	1	None	Dead (cerebral hemorrhage)
5	AAR + Sun’s procedure^*^	8	19.7	12	1000	0	None	Alive
6	AAR + Sun’s procedure	10	17.3	16	1800	2	Rupture of descending thoracic aorta, tracheotomy	Dead
7	Bentall + Sun’s procedures	5	20.3	16	1400	0	Bleeding reoperation tracheotomy	Alive
8	AAR + Sun’s procedure	2	20.5	0	200	0	None	Alive
9	Aortic pseudoaneurysm repair	13	19	8	400	0	None	Dead (infection)
10	AAR + arch replacement	3	20.6	10	0	0	None	Alive
11	AAR + Sun’s procedure	9	18.4	16	1200	0	None	Alive

AAR=ascending aortic repair; AVR=aortic valve replacement; HCA=hypothermic circulatory arrest; MVR=mitral valve replacement

* Sun’s procedure: total aortic arch replacement and frozen elephant trunk implantation

## RESULTS

The average interval between the patients’ previous operation and their last operation was 7.4±6.2 years. Two patients required emergency surgery; the other patients underwent elective operations. Operations were successfully completed in all patients. The mean intraoperative hypothermic circulatory arrest time was 8.8±7.1 minutes, and mean rectal temperature was 17.8±1.9ºC. Complications comprised one case of bleeding reoperation and two cases of postoperative tracheotomy. There were no postoperative cerebrovascular complications, paraplegia, low cardiac output, or other complications. Two patients died during hospitalization: one due to sudden refractory arrhythmia four days after operation, and the other from active rupture and hemorrhage of the descending thoracic aorta one month after operation. After discharge, the mean follow-up time was 23.4 months (range: 5-43 months), and there were two deaths. One patient died of a sudden intracerebral hemorrhage, and the other died of an infection. Of the remaining seven patients, two required thoracic endovascular aortic repair surgery at a later stage, and one developed high fever and *Staphylococcus aureus* in blood cultures one year after surgery. In the latter case, anti-infective treatment was administered for six weeks, and no fever occurred in the past year. All of these seven patients have recovered well up until the time of this writing (Table 2).

## DISCUSSION

For cardiac surgeons, redo aortic surgery is still a great challenge. One study reports that in 2555 patients who underwent redo cardiac surgery, compared with patients without injury during sternotomy, patients with injury had a higher early mortality (6.5% and 18.6%, respectively^[1]^), reducing the rate of redo aortic surgery. Complications and mortality associated with redo aortic surgery are urgent problems for cardiac surgeons.

In our center, all patients undergoing reoperation of the aorta underwent preoperative CTA to assess the risk of aortic rupture and hemorrhage during sternotomy, and to prepare relevant surgical plans. Studies have reported the importance of accurate preoperative assessment^[3]^. In this study, all patients were prepared for femoral vein cannulation before surgery, and for patients in which sternotomy hemorrhage could not be avoided, we used early cooling with CPB and reduced flow, followed by open sternotomy. It has been shown that preoperative femoral artery cannulation and sternotomy after femoral arteriovenous diversion can significantly reduce the risks associated with surgery and benefit patients^[4,5]^.

In cases of hemorrhage during sternotomy in redo aortic surgery, we currently use CPB for rapid cooling and circulatory arrest; the effect is satisfactory, with no cerebral complications and no paraplegia. Studies show that it is safe to stop the circulation for 15 minutes at medium and low temperatures (20.1-28ºC) and for 30 minutes at deep and low temperatures (14.1-20ºC)^[6]^. Svensson^[7]^ performed a series of examinations on the use of DHCA in 616 patients undergoing aortic surgery and found a stroke rate of 7% and a mean DHCA time of 31 minutes (range: 7-120 minutes). Statistical analysis showed that DHCA times > 45 minutes and > 60 minutes were independent predictors of stroke and early mortality, respectively. Atik^[8]^ summarized femoral arteriovenous diversion and used cooling with low flow or deep hypothermia to stop circulation during sternotomy, and no cases of sternotomy hemorrhage were found. For redo aortic surgery in patients with preoperative aortic valve insufficiency, if there is a risk of rupture after sternotomy, transapical left ventricular drainage should be performed in advance, so that the myocardium can be better protected during the process of circulatory arrest. Wakefield^[9]^ reported a patient with severe aortic valve insufficiency who underwent redo surgery and successful left ventricular apical drainage to prevent ventricular dilatation and dysfunction. Percutaneous intra-neck puncture was successfully used to place a retroperfusion catheter through the coronary sinus in the hybrid operation room, and an aortic balloon was placed through one side of the femoral artery for occlusion to avoid hypothermic circulatory arrest^[9]^. Mehta^[10]^ reported patients with retrosternal aortic aneurysm by percutaneous cardioplegic arrest before repeat sternotomy to avoid hypothermic circulatory arrest. However, these methods are complicated and require multidisciplinary cooperation, which is not suitable for most hospitals.

Regional development has been unbalanced in China, and the volume of redo aortic surgery tends to increase. At present, our center mainly adopts femoral arteriovenous cannulation, rapid cooling, and hypothermic circulatory arrest for arterial hemorrhage during sternotomy in redo aortic surgery. The use of MHCA or DHCA should be determined based on the size of the rupture, difficulty of repair, and surgical method utilized. If complicated with aortic regurgitation, and depending on the situation, apical intubation for left ventricular drainage should be prepared. In the future, hemorrhage during sternotomy in redo aortic surgery could be avoided with hypothermic circulatory arrest, which is the goal of cardiac surgeons.

### Limitations

This study had some limitations. First, a small number of patients were reported. Second, this was a retrospective study, and the data were obtained from a single institution.

## CONCLUSION

According to our clinical result of retrospective cases, we believe that the femoral-femoral bypass with hypothermic circulatory arrest technique is a safe and reliable method to use in cases of sternotomy hemorrhage during redo aortic surgery.

## Abbreviations, Acronyms & Symbols

AAA: Ascending aortic aneurysms
AAR: Ascending aortic repair
AD: Aortic dissection
AI: Aortic insufficiency
AV: Aortic valve
AVR: Aortic valve replacement
BMI: Body mass index
CPB: Cardiopulmonary bypass
CTA: Computed tomography angiography
DHCA: Deep hypothermic circulatory arrest
DM: Female
HCA: Hypothermic circulatory arrest
HF: Heart failure
M: Male
MHCA: Moderate hypothermic circulatory arrest
MI: Mitral insufficiency
MVR: Mitral valve replacement
TEVAR: Thoracic endovascular aortic repair
